# Supplementary material for: Retention in Care, Mortality, Loss-to-Follow-Up, and Viral Suppression among Antiretroviral Treatment-Naïve and Experienced Persons Participating in a Nationally Representative HIV Pre-Treatment Drug Resistance Survey in Mexico
Source: Pathogens. 2021 Dec 1;10(12):1569. doi: 10.3390/pathogens10121569 (PMC8706073; doi:10.3390/pathogens10121569)
Supplement: Supplementary file 1 [file pathogens-10-01569-s001.zip › pathogens-1463828-supplementary.pdf]

Supplementary Material

Table S1. Clinical outcomes according to availability of viral load information in Mexican persons living with HIV, 2017-2019.

	Outcome	experienced +resistant n=20		experienced +non-resistant n=211		naïve +resistant n=165		naïve +non-resistant n=1427		Total N=1823	
		N	VS, n(%)[1]	N	VS, n (%)	N	VS, n(%)	N	VS, n(%)	N	VS, n (%)
With viral load Information	In Care	10	5 (50%)	128	82 (64%)	118	91 (77%)	1003	843 (84%)	1259	1021 (81%)
	LTFU	3	0 (0%)	44	8 (18%)	27	9 (33%)	256	77 (30%)	330	94 (28%)
	Death	1	0 (0%)	7	2 (28%)	5	0 (0%)	35	9 (26%)	48	11 (23%)
	Total	*14 (70%)*		*179 (85%)*		*150 (91%)*		*1294 (91%)*		*1637 (89%)*	
Without viral load information	In Care	1		4		0		12		17	
	LTFU	3		18		13		81		115	
	Death	2		10		2		40		54	
	Total	*6 (30%)*		*32 (15%)*		*15 (9%)*		*133 (9%)*		*186 (10%)*	

VS, viral suppression: last viral load <200 copies/ml at the end of follow-up among persons with viral load information available within 6 months prior to the last database entry. [1]n(%): number and percentage of persons with viral suppression in each outcome and availability of vira load information status. LTFU, lost to follow-up: defined as persons with a non-active status due to ART abandonment, migration to other healthcare systems, unknown status, as well as lack of viral load follow-up for more than 6 months at the dataset closure date.

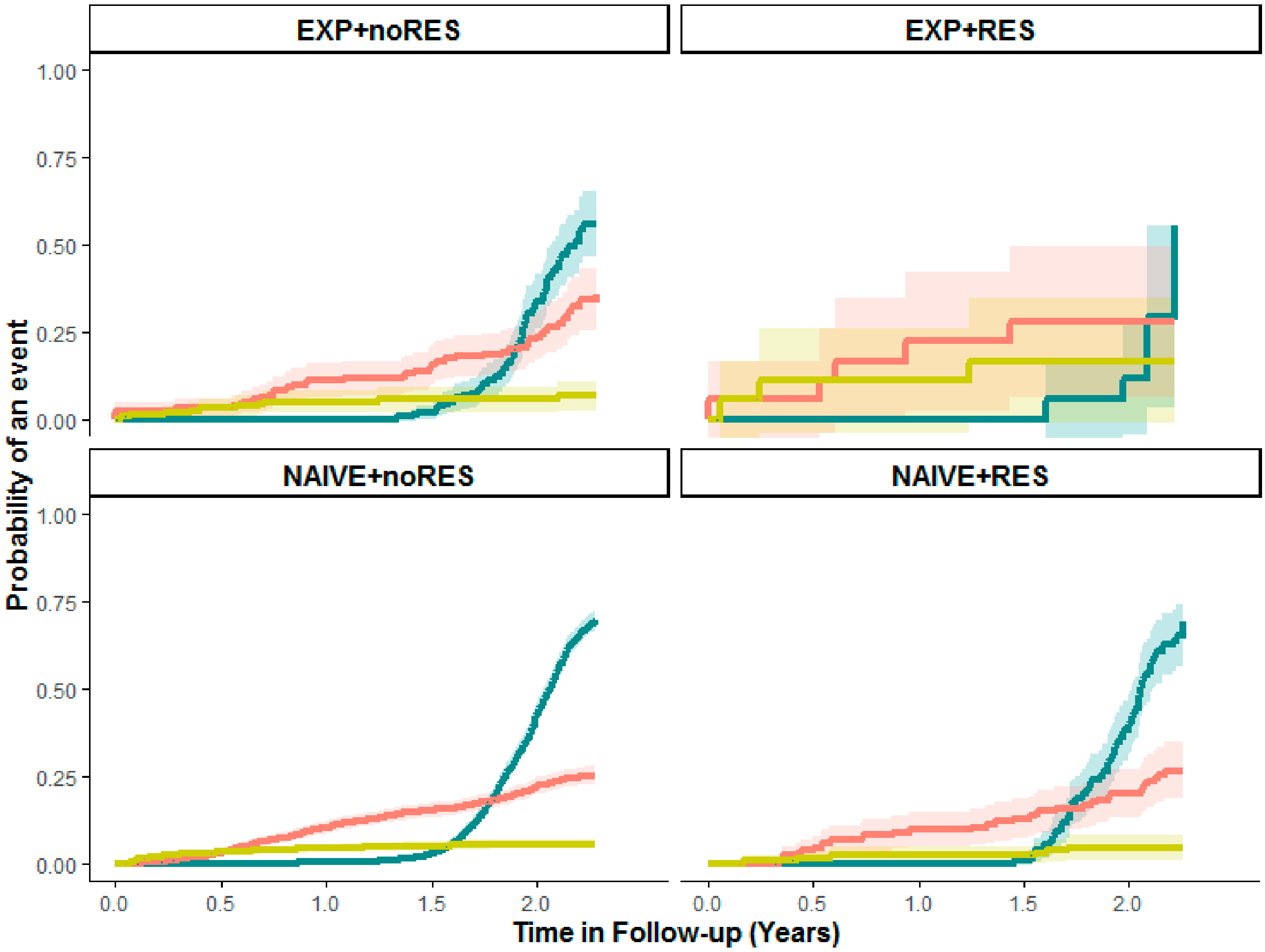

Figure S1. Estimated probability for remaining in care under viral suppression in competing risk with death and LTFU.

Note: Groups according to prior ART exposure and presence of NNRTI-PDR: EXP+noRES: experienced+non-resistant, EXP+RES: experienced+resistant, NAIVE+noRES: naive+non-resistant and NAIVE+RES: naive+resistant. Event: Viral suppression (dark green): participants with last viral load <200 copies/ml at the end of follow-up among persons with viral load information available within 6 months prior to the last database entry. LTFU, lost to follow-up (pink): defined as persons with a non-active status due to ART abandonment, migration to other healthcare systems, unknown status, as well as lack of viral load follow-up for more than 6 months at the dataset closure date. Death (ligth green).
Estimations from Fine Gray competing risk model are shown according to prior exposure to antiretroviral treatment+efavirenz/nevirapine resistance groups. The event of staying in care without viral suppression was censored. viral suppression: last viral load <200 copies/ml at the end of follow-up among persons with viral load information available within 6 months prior to the last database entry. LTFU, lost to follow-up: defined as persons with a non-active status due to ART abandonment, migration to other healthcare systems, unknown status, as well as lack of viral load follow-up for more than 6 months at the dataset closure date.
